# Transcriptional Regulation in Non-Alcoholic Fatty Liver Disease

**DOI:** 10.3390/metabo10070283

**Published:** 2020-07-09

**Authors:** Sandra Steensels, Jixuan Qiao, Baran A. Ersoy

**Affiliations:** Joan & Sanford I. Weill Department of Medicine, Weill Cornell Medical College, New York, NY 10021, USA; sas2906@med.cornell.edu (S.S.); jiq2001@med.cornell.edu (J.Q.)

**Keywords:** non-alcoholic fatty liver disease, non-alcoholic steatohepatitis, transcription factors, inflammation, metabolic stress, fibrosis, lipid homeostasis, glucose homeostasis

## Abstract

Obesity is the primary risk factor for the pathogenesis of non-alcoholic fatty liver disease (NAFLD), the worldwide prevalence of which continues to increase dramatically. The liver plays a pivotal role in the maintenance of whole-body lipid and glucose homeostasis. This is mainly mediated by the transcriptional activation of hepatic pathways that promote glucose and lipid production or utilization in response to the nutritional state of the body. However, in the setting of chronic excessive nutrition, the dysregulation of hepatic transcriptional machinery promotes lipid accumulation, inflammation, metabolic stress, and fibrosis, which culminate in NAFLD. In this review, we provide our current understanding of the transcription factors that have been linked to the pathogenesis and progression of NAFLD. Using publicly available transcriptomic data, we outline the altered activity of transcription factors among humans with NAFLD. By expanding this analysis to common experimental mouse models of NAFLD, we outline the relevance of mouse models to the human pathophysiology at the transcriptional level.

## 1. Introduction

Obesity often results in the dysregulation of lipid and glucose metabolism and is therefore the primary risk factor for the pathogenesis of metabolic disorders, including cardiovascular disease, type 2 diabetes mellitus (T2DM), and non-alcoholic fatty liver disease (NAFLD) [1]. The global prevalence of NAFLD, which was 15% in 2005, has quickly escalated to 24% by 2016 in a parallel trend to obesity [2]. NAFLD encompasses a spectrum of pathologies ranging from hepatocellular lipid accumulation (steatosis) to non-alcoholic steatohepatitis (NASH) characterized by steatosis and inflammation. In addition, chronic inflammation activates hepatic stellate cells (HSC), which promote fibrosis by secreting type I and III collagen and fibronectin into the extracellular matrix (ECM) [3]. When fibrotic NASH remains untreated, it can lead to cirrhosis and hepatocellular carcinoma (HCC) [4]. Despite alarming increases in prevalence, the treatment strategy of NAFLD remains limited to weight loss regiments and requires a more complete understanding of diet-induced pathogenesis of NAFLD in obese patients [5].

The pathogenesis of NAFLD is complex, and evolving theories have culminated in a two-hit versus multiple-hit hypotheses [6]. In the ‘two-hit hypothesis’, the first hit originates from the accumulation of more than 5% hepatic steatosis, during which insulin resistance emerges as a pathogenic contributor. This makes the liver more susceptible to a second hit, including oxidative stress, the production of pro-inflammatory cytokines, and apoptosis, which progress the disease to the necro-inflammatory stage defined as NASH [7]. In contrast, the ‘multiple-hit’ hypothesis encompasses the interplay of multiple factors whereby genetics, environment, unhealthy dietary habits, insulin resistance, adipocyte differentiation, and the intestinal microbiota together contribute to disease development and progression [8]. Regardless of the source of the hit (s), hepatic responses to extrahepatic stimuli are controlled by well-described transcriptionally regulated pathways that help transcribe the relevant biological machinery to maintain energy homeostasis. However, obesity-induced maladaptive activation or the inhibition of these transcriptional regulators often exacerbates lipid accumulation, insulin resistance, inflammation, and fibrosis [9].

The efforts toward identifying the promoters of obesity-induced NAFLD have relied heavily on rodent models due to limited access to and the variability within human samples arising from differences in disease stage, age, sex, medication, body weight, and other lifestyle choices such as alcohol consumption. However, rodent models do not capture all the features of the human pathophysiology. The rodent NAFLD models described in this review are categorized by their mode of induction using diet, chemicals, or genetic alteration (Box 1). For the diet-induced models, we highlight high-fat diet (HFD), Western diet (WD), methionine- and choline-deficient diet (MCD), choline-deficient l-amino acid-defined (CDAA) diet, and fructose-palmitate-cholesterol and trans-fat (FPC or NASH) diet. The chemically induced models include the combination of HFD with streptozotocin (STZ) supplementation or the use of carbon tetrachloride (CCl_4_). For genetic models, we highlight the APOE2 knock-in (APOE2-KI) mouse [10], hepatocyte-specific phosphatase and tensin homolog (PTEN) knockout model [11], and Mice expressing urokinase-type plasminogen activator (uPA) under the control of the major urinary protein (MUP) promoter (MUP-uPA mice) [12].

Box 1 **Mouse models of non-alcoholic fatty liver disease (NAFLD).**

Diet-based models

*High-fat diet (HFD, 60 kcal% fat) and Western diet (WD, 40% kcal fat and 40% kcal carbohydrates)*—HFD feeding of mice (8–12 weeks) leads to a phenotype similar to simple steatosis in humans, which is characterized by obesity, insulin resistance, and hyperlipidemia [13]. Alanine aminotransferase (ALT) and aspartate aminotransferase (AST) levels also become exacerbated after extended exposure (>8 months). However, this diet barely induces fibrosis even after extended exposure (up to 1 year) [13].

*MCD diet*—In the MCD diet, the absence of methionine (4–8 weeks) leads to hepatic injury, inflammation, and fibrosis, while the deficiency of choline leads to macrovesicular steatosis. Due to the nature of its pathogenesis, this model is less representative of the initiation of NAFLD in humans. Nonetheless, the diet induces progressive steatohepatitis leading to fibrosis, which is histologically similar to the human disease. The main drawback of MCD is its induction of body weight loss and decrease in plasma triglyceride levels [14].

*CDAA diet*—CDAA is similar to MCD due to their shared deficiency in choline. However, in CDAA, proteins are substituted with an equivalent and corresponding mixture of l-amino acids [15]. Animals fed CDAA develop the same or perhaps a more severe degree of NASH as well as a larger increase in alanine aminotransferase (ALT) levels, albeit on a longer time frame (12 weeks) [15].

*FCP (NASH) diet*—The FCP or NASH diet entails a HFD supplemented with 1.25% cholesterol and drinking water containing glucose and fructose (95%/45%, *w*/*v*). The FCP diet includes Western and American Lifestyle-Induced Obesity Syndrome model diets to achieve both metabolic and hepatic NASH features within 4 months. Fructose-supplemented drinking water for eight weeks results in simple steatosis in rodents without features of NASH and induces a significant increase in body weight and plasma triglyceride and glucose levels [16].

Pharmacological models

*STAM*—STZ-induced T2DM is a well-known experimental model of T2DM and is achieved by the administration of a low dose of STZ shortly after birth, which results in the apoptotic death of insulin-secreting pancreatic islets. When this approach is combined with HFD, it can be used as a model for NAFLD and NASH [17]. This model results in simple steatosis at 6 weeks of age, NASH with inflammatory foci and ballooning at 8 weeks, and progressive peri-cellular fibrosis starting between 8 and 12 weeks. Starting at 6 weeks of age, mice exhibit elevated ALT levels and fasting glycemia. Multiple hepatocellular carcinomas appear after 20 weeks of treatment [17].

*CCl_4_*—Supplementation of diet with CCl_4_ exacerbates the histological features of NASH, fibrosis, and tumor development in the setting of HFD. HFD coupled with CCl_4_ results in advanced fibrosis at 12 weeks and HCC at 24 weeks in rodent models [18].

Genetic models

*Apoe*—A rodent model that replicates the early stages of NAFLD is the APOE2-KI mouse in which the mouse *Apoe* gene is replaced by the human APOE2 allele. In addition to dyslipidemia and atherosclerosis, APOE2-KI mice develop diet-induced NASH when fed WD. A major advantage of this mouse model is that it displays good responses to pharmacological treatments [10].

*Pten*—PTEN is a tumor suppressor gene mutated in many human cancers, and its expression is reduced or absent in almost half of hepatoma patients, making this a relevant model for human HCC [11]. Hepatocyte-specific PTEN deficiency results in steatohepatitis and HCC in mouse models [11].

*MUP-uPA mice*—This model is based on feeding HFD to MUP-uPA transgenic mice, which express high amounts of uPA specifically in hepatocytes during the first 6 weeks of life [12]. HFD-fed MUP-uPA mice exhibit increased HSC activation and a substantial upregulation of collagen gene expression. Key diagnostic parameters of NASH, including ballooning, inflammatory infiltrates and pericellular and bridging fibrosis, are evident following 4 months of HFD and are indistinguishable from human NASH, making this a relevant study model [12].

In this review, we discuss our current understanding of the transcription factors that have been linked to the pathogenesis and progression of NAFLD. Transcription factors that are associated with obesity-induced liver injury and the pathogenesis and progression of NAFLD often serve essential biological functions in the maintenance of energy homeostasis and stress response. Furthermore, recent studies have indicated that the gut microbiota may contribute to NAFLD by altering the production of endogenous substrates that control the activity of hepatic transcription factors. Therefore, we have categorized these transcriptional regulators under lipid and glucose metabolism, inflammation, metabolic stress, fibrosis, and microbiome dysbiosis. Key transcriptional regulators that play significant roles in multiple metabolic responses have been addressed in all relevant categories.

## 2. Lipid Metabolism

Hepatic steatosis is a consequence of increased hepatic lipid uptake, increased *de novo* lipogenesis, and reduced lipid clearance. Excessive nutrition, accompanied by hyperinsulinemia and hyperglycemia, drives steatosis by promoting *de novo* lipogenesis in the liver, which contributes substantially to the accumulation of triglycerides and other lipid species [19]. Hepatic lipid homeostasis is mainly regulated by peroxisome proliferator-activated receptor alpha (PPARα), PPARγ, PPARδ and sterol regulatory element binding protein 1c (SREBP1c), which coordinate transcriptional responses to altered metabolic conditions such as feeding and fasting to promote fat storage or catabolism, respectively. Other transcription factors of lipid metabolism that are altered in the setting of NAFLD include the constitutive androstane receptor (CAR), liver X receptor (LXR), Cyclic AMP-responsive element-binding protein H (CREBH), Farnesoid X receptor (FXR), signal transducer and activator of transcription 5 (STAT5), and CCAAT/enhancer binding protein alpha (C/EBPα) (Table 1). 

### 2.1. PPARα

PPARα belongs to the PPAR nuclear receptor family. PPARα is mostly expressed in hepatocytes where it becomes activated upon binding by fatty acids (FAs) and promotes FA uptake and utilization through β-oxidation and ketogenesis [46]. Hepatic PPARα expression is increased in male mice and both male and female humans with NAFLD [20,46]. Suggestive of a protective function, mice lacking PPARα expression exhibit more severe steatosis [47]. Therefore, NAFLD-induced increases in PPARα abundance can be further enhanced by its pharmacological activation: the PPARα agonist WY-14643 protects mice against steatosis and steatohepatitis by preventing intrahepatic lipid and lipoperoxide accumulation [47]. Since WY-14643 causes toxicity in humans, other fibrates have been extensively used in the treatment of hypertriglyceridemia. However, these studies failed to establish benefits against NASH, which is most likely due to the widespread extrahepatic expression of PPARα [48].

### 2.2. PPARγ

Another member of the PPAR family, PPAR**γ** is also activated by FA ligands and promotes lipogenesis and lipid accumulation. In humans and mice, two isoforms of PPAR**γ** exist: PPAR**γ**1 is found in nearly all tissues except muscle, while PPAR**γ**2 is mostly expressed in adipose tissue and the intestine. PPAR**γ**2 expression is upregulated in the liver and adipose tissue of obese humans and high-fat diet (HFD)-fed mice, whereas the PPAR**γ**1 expression remains unchanged under these conditions [49]. In hepatocytes, PPAR**γ**1 increases the transcription of genes that are required for FA uptake and *de novo* lipogenesis [50]. Meanwhile, lipidomic analyses suggest that PPAR**γ**2 plays an important anti-lipotoxic role when induced ectopically in liver and muscle by facilitating the deposition of lipid droplets and preventing the accumulation of reactive lipid species, such as ceramides and pro-inflammatory lysophosphatidylcholine [51]. HFD-fed mice with a hepatocyte specific loss of PPAR**γ** expression exhibit a reduction of hepatic lipid vacuoles as well as the downregulation of genes involved in *de novo* lipogenesis [52]. Furthermore, the liver-specific ablation of PPAR**γ** in *ob*/*ob* mice reduces hepatic triglycerides despite increasing serum FAs [53]. Livers of NAFLD patients have increased hepatic PPARγ expression [20,46]. Whereas increased PPARγ activity within hepatocytes would be expected to contribute to steatosis [54,55], the treatment of patients with the PPARγ agonists rosiglitazone or pioglitazone result in reduced hepatic steatosis [56,57,58]. This alleviation could be explained by the extrahepatic effects of PPARγ activation in the adipose tissue where it promotes the storage of excess energy in the form of lipid droplets, thereby limiting exposure of the liver to excess lipids. 

### 2.3. PPARδ

Similar to other PPARs, PPARδ binds to the PPAR response element (PPRE) to initiate or repress the expression of target genes [59]. PPARδ is ubiquitously expressed and is activated by polyunsaturated fatty acids and their metabolites. In mouse livers, PPARδ prevents lipid accumulation by increasing β-oxidation and autophagy. In addition, the activation of PPARδ in the adipose tissue of mice upregulates the expression of genes involved in β-oxidation and energy dissipation [60,61]. Recent clinical studies using PPARδ agonists atorvastatin and cardarine reduced hepatic fat content in overweight patients with mixed dyslipidemia [61,62].

### 2.4. SREBP

The SREBP family transcription factors consist of three isoforms: SREBP1a, SREBP1c, and SREBP2. Each isoform exhibits a different tissue expression pattern and metabolic control [63]. SREBP1a is the predominant isoform in the intestine, spleen, and cultured cells, while SREBP1c and SREBP2 exhibit higher abundance in the liver [63]. SREBP1a is a potent activator of genes that mediate the synthesis of cholesterol, fatty acids, and triglycerides. The roles of SREBP1c and SREBP2 are more restricted than those of SREBP1a. SREBP1c promotes the transcription of genes involved in lipogenesis, such as acetyl-coenzyme A (CoA) carboxylase (ACC), FA synthase (FASN), and steroyl–CoA desaturase in response to insulin and high-energy state [64]. By contrast, hepatic markers for energy deprivation, such as glucagon signaling (protein kinase A [PKA], AMP activated protein kinase [AMPK]) and the deacetylase sirtuin1 (SIRT1) inhibit SREBP1c, suggesting that SREBP1c does not promote hepatic lipid synthesis in the setting of starvation [65]. Among the genes involved in lipogenesis, SREBP1c also promotes the transcription of patatin-like phospholipase3 (PNPLA3), which in turn stimulates lipid accumulation [66]. Independent studies in humans have confirmed that PNPLA3 variants are strongly associated with the severity of NAFLD and NASH [67,68,69]. SREBP1c is upregulated in the livers of humans and mice with NAFLD [70]. Interestingly, there is also a positive correlation between single nucleotide polymorphisms (i.e., rs2297508) as well as rare variants of *SREBP1* with the risk of developing NAFLD [21]. Unlike SREBP1c, SREBP2 preferentially activates cholesterol synthesis [71]. In mice, SREBP2 contributes to the onset of NASH by triggering cholesterol accumulation [72]. Increased hepatic SREBP2 is also associated with increased free cholesterol in NASH patients [73].

### 2.5. CAR

CAR is a member of the nuclear receptor superfamily [74]. It mainly functions as a sensor of endobiotic and xenobiotic substances, as CAR-activated genes regulate drug metabolism and enhance bilirubin clearance [74]. Unlike most nuclear receptors, this transcriptional regulator is constitutively active in the absence of a ligand. CAR activity is anti-obesogenic and improves insulin sensitivity [75]. The metabolic benefits of CAR activation stem from the combined effects of reduced lipogenesis, very low-density lipoprotein (VLDL) secretion, and gluconeogenesis, as well as increased peripheral fat mobilization for thermogenesis in brown adipose tissue [75]. The anti-steatotic effect of CAR was first demonstrated using a mouse model with the genetic ablation of cytoplasmic CAR retention protein (CCRP), which isolates CAR to the cytosol and inactivates it. Subsequent CAR activation represses lipogenic gene expression and increases β-oxidation [76]. Similar to mouse models of NAFLD, CAR is also upregulated in the livers of patients with NAFLD [23].

### 2.6. LXR

LXR is a member of the nuclear receptor family of transcription factors that is closely related to PPARs [77]. LXR forms heterodimers with the obligate partner retinoid X receptor (RXR), which is activated by retinoic acid and cholesterol derivatives. LXR is an important regulator of cholesterol, FA, and glucose homeostasis [77]. LXR activation increases hepatic triglyceride accumulation and cholesterol metabolism in both humans and mice and initiates bile acid degradation in mice [78]. Humans express two LXR family members, namely LXRα (*NR1H3*) and LXRβ (*NR1H2*). LXRα expression increases by 2- and 3-fold in the livers of NAFLD and NASH patients, respectively, compared to healthy controls [24]. Furthermore, LXRα expression positively correlates with the amount of hepatic fat and hepatic expression of the cholesterol transporter ATP-binding cassette sub-family G member 5 (ABCG5/8), the FA transporter cluster of differentiation 36 (CD36), and SREBP1c [24]. 

### 2.7. CREBH

CREBH is primarily expressed in the endoplasmic reticulum (ER) of cells in the liver and small intestine [79,80]. CREBH expression increases in response to fasting through glucagon signaling [81]. CREBH expression is also controlled by the binding of glucocorticoid or PPRE to its promotor region [82]. Therefore, CREBH expression can be induced by a variety of PPARα agonists such as palmitate and oleate [82]. ER-anchored CREBH becomes activated in response to hepatic lipid accumulation and VLDL assembly. The activation of CREBH requires ER-to-golgi trafficking followed by proteolytic cleavage and nuclear translocation [81,83,84]. CREBH activates a group of genes that are involved in TG and lipoprotein production [85,86]. CREBH also binds to and functions as a co-activator for both PPARα and LXRα to promote FA uptake and utilization [86]. CREBH-deficient mice are susceptible to hepatic steatosis following fasting [81] or diets with high-fat content [79,86]. Interestingly, the livers of CREBH-deficient mice exhibit the reduced expression of genes that promote *de novo* lipogenesis and FA elongation [86]. Observed steatosis most likely arises from the reduced hepatic expression of genes involved in FA oxidation and increased lipolysis in the adipose tissue, resulting in an increased flow of FA from adipose tissue to the liver [79]. Furthermore, fibroblast growth factor (FGF) 21 is a critical CREBH target that reduces hepatic lipid storage. CREBH overexpression in the livers of mice suppresses hepatic lipid accumulation by increasing FGF21 secretion [87].

### 2.8. FXR

FXR is a major member of the ligand-activated nuclear receptor superfamily [78]. The family consists of four isoforms namely, FXRα1, FXRα2, FXRβ1, and FXRβ2 [88]. Similar to LXR, bile acids are natural ligands for FXR, which plays an important role in regulating bile acid homeostasis, glucose and lipid metabolism, intestinal bacterial growth, and hepatic regeneration [89]. While LXR facilitates the storage of carbohydrate- and fat-derived energy, FXR decreases TG levels and improves glucose metabolism [90]. One of the primary functions of FXR activation is the suppression of CYP7A1, the rate-limiting enzyme in bile acid synthesis from cholesterol [91]. FXR expression is decreased in NASH patients [25], which can aggravate the development of steatosis and NASH: (1) FXR activation represses hepatic lipogenesis via the FXR–SHP–SREBP1c pathway (see below for more on small heterodimer partner [SHP]), (2) FXR activation promotes β-oxidation by stimulating the expression of PPARα and CPT1, and (3) FXR activation reduces hepatic FA uptake by reducing the expression of CD36 [89]. 

### 2.9. STAT5

STAT5 belongs to a family of intracellular transcription factors that are activated by membrane receptor-associated Janus kinases (JAK). The growth hormone (GH)-mediated activation of STAT5 [92] plays an important role in hepatic fat metabolism through the downregulation of CD36 [93]. The liver-specific loss of STAT5 in mice induces hepatic steatosis following a HFD [92]. These mice also exhibit hyperglycemia, hyperinsulinemia, hyperleptinemia, and elevated free FA and cholesterol concentrations following HFD. At the transcriptional stage, the loss of STAT5 results in the transcription of genes involved in lipid uptake (CD36), VLDL uptake (very low-density lipoprotein receptor), and lipogenesis (stearoyl-CoA desaturase and PPARγ) [93]. However, it is unclear whether STAT5 directly regulates the expression of these factors. In addition, its relevance in human steatosis associated with GH-deficiency is yet to be established.

### 2.10. C/EBPα

C/EBPα belongs to a transcription factor family of six members which are involved in a variety of cellular responses [94]. C/EBPα plays a role in lipogenic gene expression by inducing the expression of PPARγ [95]. The liver-specific ablation of C/EBPα reduces lipogenic gene expression and triglycerides in the livers of leptin-deficient *ob*/*ob* mice, which otherwise display severe steatosis [95]. These findings were confirmed by a similar observation of reduced hepatic gene expression following siRNA-mediated inhibition of C/EBPα expression in the livers of leptin receptor-deficient (*db*/*db*) mice [27].

## 3. Glucose Metabolism

The liver does not only play a central role in systemic lipid homeostasis but also regulates the glucose balance in circulation. This is mediated by the activation of carbohydrate-responsive element-binding protein (ChREBP) in response to increases in plasma glucose and the nuclear localization of PPARγ coactivator 1 alpha (PGC1α), cAMP response element binding protein (Creb), CREBH, forkhead protein O1 (FOXO1), and hepatocyte nuclear factor 4α (HNF4α) in response to fasting to promote hepatic glucose production [96] (Table 1). Furthermore, PPARδ also plays a role in glucose homeostasis. The exacerbation of hepatic glucose production coupled with hyperglycemia and insulin resistance play an important pathogenic role in NAFLD. 

### 3.1. ChREBP

ChREBP consists of ChREBPα, the full-length isoform, or ChREBP-β, the truncated isoform [97]. ChREBPα is directly activated by glucose, independently from insulin signaling [98]. Little is known about ChREBPβ, which was reported to be expressed in a glucose- and ChREBP-dependent manner whereby glucose-activated ChREBPα initiates ChREBPβ transcription from an alternate promoter [99]. In the liver, ChREBP promotes glycolysis and lipogenesis. ChREBP expression is increased in the livers of NASH patients with advanced steatosis [100]. By contrast, decreased ChREBP expression is associated with severe insulin resistance [22]. This pattern indicates that ChREBP is essential for the storage of excess glucose as triglycerides. In fact, mice that overexpress ChREBP exhibit improved insulin sensitivity and glucose tolerance despite having more pronounced hepatic steatosis. Together, these studies have demonstrated that increased ChREBP activity improves insulin sensitivity by promoting simple steatosis without lipotoxicity [22]. 

### 3.2. PGC1α

The PGC1 family of transcriptional co-activators play a central role in the regulation of metabolism. The PGC1 family consists of three members, namely PGC1α, PGC1β, and the PGC-related co-activator (PRC), which interact with transcription factors and nuclear receptors to exert their biological functions. PGC1α expression is induced by metabolic cues such as exercise, cold, and fasting [101]. The activation of PGC1α in the liver drives the expression of genes that are essential to gluconeogenesis, FA oxidation, lipid transport, and mitochondrial biogenesis. The activity of PGC1α becomes impaired in the setting of liver injury and steatosis in mice, and the loss of PGC1α has been linked to the increased susceptibility to NAFLD in HFD-fed mice [102]. PGC1α haploinsufficiency in mouse liver inhibits β-oxidation and increases triglyceride synthesis, leading to hepatic steatosis and insulin resistance. Similarly, PGC1α overexpression in rat hepatocytes results in reduced concentrations of hepatic triglycerides in vitro and in vivo, due to increased β-oxidation [28]. 

### 3.3. CREB

CREB becomes activated in response to glucagon-mediated increases in cellular cAMP. The knockdown of CREB dramatically reduces fasting plasma glucose concentrations in several rodent models for obesity and type 2 diabetes, including Zucker diabetic fatty (ZDF) rats, STZ-treated/HFD-fed rats, and *ob*/*ob* mice. CREB does not only promote the expression of gluconeogenic genes but also increases plasma TG and cholesterol concentrations as well as hepatic steatosis by activating *de novo* lipogenesis in the liver [103]. 

### 3.4. CREBH

CREBH was reported to bind and upregulate genes that contain cAMP-responsive elements, including phosphoenolpyruvate carboxykinase 1 (*Pck1*) and glucose-6-phosphatase (G6Pase) [80,81], which are essential promoters of gluconeogenesis. CREBH also upregulates the rate-limiting enzyme for hepatic glycogenolysis, namely glycogen phosphorylase (*Pygl*) [81]. Consequently, CREBH overexpression in the livers of mice increases plasma glucose levels, while its knockdown reduces circulating glucose [81].

### 3.5. FOXO

The forkhead protein family comprises of more than 100 members in humans and are enumerated FOXA to FOXR based on their sequence similarity [104]. The members of the FOXO subfamily, which consists of FOXO1, FOXO3, FOXO4, and FOXO6, are regulated by insulin signaling whereby Akt-mediated phosphorylation sequesters FOXOs within the cytosol, inhibiting their transcriptional activity in the nucleus [105]. FOXO family members mediate the expression of genes that play a role in cell death, DNA repair, glucose, and energy metabolism [106]. Hepatic FOXO1 regulates the expression of both gluconeogenic and lipogenic genes. Under fasting conditions, FOXO1 drives the expression of gluconeogenic enzymes. In addition, FOXO1 induces the transcription of genes involved in the hepatic assembly of VLDL, reducing hepatic steatosis [106]. The genetic ablation of FOXO increases susceptibility to NAFLD and NASH in mice [105]. Specifically, the deletion of FOXO1/3 or FOXO1/3/4 genes in mouse livers leads to mild or moderate hepatic steatosis, even when mice are maintained on a regular chow diet [105]. Exposing the mice to HFD supplemented with cholesterol further exacerbates steatosis in FOXO1/3/4-deficient mice [105]. Conversely, the overexpression of a constitutively active FOXO1 reduces hepatic triglycerides [105]. On the other hand, livers of NASH patients exhibit a greater expression of FOXO1 compared to patients with simple steatosis as well as metabolically healthy patients with and without obesity [29]. More investigation into FOXO1 activity during different stages of human liver disease is needed to establish mechanisms and physiological relevance. 

### 3.6. HNF4α

HNF4α is a member of the nuclear hormone receptor superfamily and has been shown to play an essential role in maintaining bile acid, lipid, and glucose homeostasis. HNF4α translates extracellular endocrine signals and intracellular stress and nutritional state onto transcriptional responses in the liver. HNF4α is regulated by growth hormone, glucocorticoids, thyroid hormone, insulin, transforming growth factor beta (TGFβ), estrogen, and cytokines [107]. HNF4α target genes have been identified in the liver, pancreas, and colon. In the liver, the targets include genes involved in glucose (PEPCK, glucose-6-phosphatase (G6Pase)), bile (CYP7A1), xenobiotics and drug metabolism (CYP3A4, CYP2D6, and CYP2E1) [108]. HNF4α also regulates circulating levels of cholesterol and triglycerides by inducing the transcription of genes that encode for apolipoproteins. HFD-induced oxidative stress promotes hepatic steatosis by blocking the activity of HNF4α in mice [109]. The expression of HNF4α is decreased in NASH patients [25]. Furthermore, a systematic integrative analysis of gene transcription has identified HNF4α as ‘the central gene’ in the NASH pathogenesis [25].

### 3.7. PPARδ

In addition to its activation of fatty acid oxidation, PPARδ improves glucose homeostasis and protects from insulin resistance by promoting insulin secretion in the pancreatic islet β-cells [110,111] and by increasing energy utilization [112]. Mice lacking PPARδ expression have reduced energy expenditure and are glucose-intolerant. In contrast, receptor activation by GW50516, a PPARδ-specific agonist, suppresses hepatic glucose output, improves insulin sensitivity and increases glucose disposal in mice [112]. This increase in energy disposal has been linked to increased β-oxidation in the skeletal muscle of mice following GW50516 treatment [113].

## 4. Inflammation

The hepatic inflammatory response is an important driving force for NASH progression as it promotes sustained hepatic fibrogenesis. Transcription factors activated in response to inflammatory stimuli mainly belong to the family of nuclear factor of the κ light chain enhancer of B cells (NF-κBs), interferon regulatory factors (IRFs), STAT, and activator protein 1 (AP-1) [114]. Other factors that have also been implicated in the transcriptional regulation of the inflammatory response include apoptosis antagonizing transcription factor (AATF, synonym: che-1), SHP, Runt-related transcription factor 2 (Runx2), and C/EBPβ. In addition to inflammation-specific regulators, transcriptional regulators of lipid homeostasis PPARα, PPARγ, CAR, and LXR also affect the hepatic inflammatory state (Table 1). 

### 4.1. NF-κB

NF-κB is a protein complex that controls cytokine production and cell survival, and as such, it plays a key role in the immune response to infection. NF-κB is also critical for the development of inflammation in various metabolic disorders such as T2DM [115] and is highly activated in both mice and patients with NASH [30,116]. The pharmacological inhibition of NF-κB signaling protects MCD-fed mice from the pathogenesis of NASH with significant reductions in hepatocellular injury and hepatic inflammation. Furthermore, the stage of inflammation and fibrosis in livers of NASH patients correlates with the expression of the p65 subunit of NF-κB [117]. 

### 4.2. IRFs

IRFs are a family of transcription factors that regulate the transcription of interferons and consist of nine members. Most IRFs are involved in innate immunity and defense against pathogens. IRF family members impose variable impacts on inflammation in the pathogenesis of NAFLD. Studies using mice with the deletion of IRF7 expression indicated that IRF7 promotes weight gain, hepatic fat deposition, and insulin resistance in the setting of HFD [118]. In contrast, a similar study using IRF9-deficient mice demonstrated that IRF9 promotes insulin sensitivity and attenuates inflammation and hepatic steatosis [119]. Interestingly, IRF9 was shown to interact with PPARα and activate its target genes [119].

### 4.3. STAT

STAT family members with inflammatory biological functions (STAT1 and STAT3) have been associated with NAFLD and NASH. The oxidative hepatic environment in obesity inhibits the STAT1 and STAT3 phosphatase, T cell protein tyro-sine phosphatase (TCPTP), which results in increased STAT1 and STAT3 signaling. This in turn increases the risk of developing NASH and HCC in the setting of excessive nutrition [32]. Furthermore, the inactivation of TCPTP, coupled with increased STAT1 and STAT3 signaling, are easily detectable events in the livers of humans with NASH [32].

### 4.4. AP1

AP1 activation requires the synthesis of c-Jun and c-Fos proteins and c-Jun phosphorylation by c-Jun N-terminal kinase (JNK) for the full transactivation of target genes. Obese patients with NASH exhibit an enhanced hepatic expression of AP1 targets [30]. JNK activation and the extent of c-Jun nuclear localization correlates very well with the pathogenesis and progression of NASH in humans and mouse models [33]. Activated c-Jun promotes nuclear accumulation of JNK, which provides a positive feedback loop to further enhance AP1 transcriptional activity and exacerbate NASH progression [120,121].

### 4.5. AATF

AATF mediates cell proliferation and survival [122,123,124]. Its expression in the liver increases with simple steatosis [44]. Indicative of a role in inflammation, AATF expression increases in response to tumor necrosis factor α (TNFα)-mediated activation of SREBP1 in cultured cells. In turn, AATF induces the expression of the inflammatory cytokine monocyte chemotactic protein 1 (MCP1) by activating STAT3. Hepatic AATF expression does not increase any further with disease progression to NASH [44], suggesting that it plays a role in exacerbating simple steatosis toward the pathogenesis of inflammatory stages of steatohepatitis. However, its contribution to the progression of NASH to advanced stages remains unclear.

### 4.6. SHP

SHP is technically not a transcription factor, since it lacks a DNA binding domain but is still classified as such due to its sequence homology to other nuclear receptor families. The principal role of SHP is the repression of other nuclear receptors by binding and forming a dysfunctional heterodimer. SHP is a critical repressor of various genes involved in glucose and lipid metabolism and bile acid synthesis [125]. Several factors indicate a role in inflammation: first, SHP inhibits inflammatory responses that are triggered by the Toll-like receptor (TLR) [126] as well as the NLR family pyrin domain containing 3 (NLRP3) inflammasome, which consists of a multimeric protein complex that triggers inflammatory cell death and the release of pro-inflammatory cytokines interleukin (IL)-1β and IL-18 [127]. In addition, SHP suppresses inflammation by inhibiting transcription of the chemokine CCL2 whose biological function is to recruit macrophages and promote inflammation [34]. The SHP-mediated mitigation of inflammatory responses could play a protective role in NASH. SHP expression is drastically decreased in the livers of a mouse model of NASH and in the livers of NASH patients compared healthy or steatotic livers [34]. The rescue of SHP expression in the livers of mice prevents the progression of NAFLD to NASH [34]. Mechanistically, the reduction of SHP expression in NASH was linked to inhibitory binding of c-Jun to the SHP promoter, suggesting that the JNK/SHP/NF-κB/CCL2 axis is a promising target for NASH prevention and treatment. 

### 4.7. Runx2

Runx2 plays an important role in atherosclerosis. It has been indicated that atherosclerosis shares a similar histopathology with NASH with respect to macrophage infiltration. Indeed, experiments in mouse primary HSCs have elucidated a mechanism whereby Runx2 within HSCs promotes macrophage infiltration by increasing the transcription of MCP1 [36]. 

### 4.8. C/EBPβ

C/EBPβ was originally identified as nuclear factor interleukin-6 (NFIL6) because of its inducibility by IL-6 and its important role in the activation of acute inflammatory response genes in human hepatoma cells [128]. The livers of mice lacking C/EBPβ express reduced markers of inflammation and endoplasmic reticulum (ER) stress and exhibit decreased steatosis following an MCD diet. By contrast, C/EBPβ overexpression increases the hepatic prevalence of PPARγ, ER stress, NF-κB activation, and steatosis [27].

### 4.9. PPARα

In addition to its role in the regulation of metabolism, PPARα also exhibits anti-inflammatory effects through its regulation of NF-κB [129]. The treatment of non-steatotic mice with the PPARα agonist WY-14643 decreases the hepatic inflammatory gene expression profile, suggesting a direct anti-inflammatory effect of PPARα independent of changes in liver triglycerides [130]. 

### 4.10. PPAR*γ*

Hepatic PPARγ does not only regulate hepatocyte metabolism but also plays an important regulatory role in liver-resident macrophages (Kupffer cells), where it acts as an inhibitor of macrophage activation and cytokine production. This regulation is mediated through the PPARγ1-mediated inhibition of AP-1, STAT, and NF-κB, which are the major regulators of macrophage activation and TNFα synthesis [131]. Mice with Kupffer cell-specific loss of PPARγ expression exhibit increased hepatic expression of inflammatory cytokines TNFα and IL1β and fibrosis in response to CCl_4_-induced liver injury [132]. Conversely, PPARγ induction by rosiglitazone decreases the number of hepatic Kupffer cells, attenuating the inflammatory response as well as steatosis in a diet-induced mouse model of NAFLD [133].

### 4.11. CAR

CAR activation can potentially be used to delay or reduce the progression of NAFLD due to its dual anti-steatotic and anti-inflammatory effects. In the MCD mouse model of NASH, the administration of the CAR agonist 2,2′-[1,4-phenylenebis(oxy)]bis[3,5-dichloro]-pyridine (TCPOBOP) reduces inflammation and hepatocellular apoptosis by reducing the accumulation of Kupffer cells and enhancing the hepatic clearance of pro-inflammatory leukotriene B4 [134]. On the other hand, CAR knockout mice exhibit improved lipid peroxidation and hepatic fibrosis after exposure to the MCD diet [135]. Therefore, the precise role of CAR in the pathophysiology of NASH requires additional studies.

### 4.12. LXR

Although LXR promotes inflammation, its impact on obesity and steatosis is inconsistent. Mouse models with the deletion of LXR expression have indicated that LXR decreases inflammation by inhibiting the transcription of TNFα, IL-6, and IL-1β but increases steatosis [136]. On the other hand, the treatment of mice with the LXR antagonist SR9238 generates both anti-steatotic [137] and anti-fibrotic effects [138] with a dramatic reduction in steatosis, inflammation, and collagen disposition in the livers of mice. Overall, all studies indicate that LXR could be a valuable target for the treatment and prevention of NASH.

## 5. Metabolic Stress

The pathogenesis of NAFLD does not only depend on energy metabolism and inflammation but has also been mechanistically linked to increased cellular stress. Upon excessive nutrition, the ER cannot meet high metabolic demands and initiates the unfolded protein response (UPR) by activating three transmembrane factors located on the ER membrane: protein kinase R-like ER kinase (PERK), inositol-requiring enzyme 1α (IRE1α), and activating transcription factor 6 (ATF6). PERK activates eukaryotic translation initiation factor 2α (eIF2α), which in turn activates ATF4. Meanwhile, IRE1α splices the mRNA of X box-binding protein 1 (Xbp1) to its active isoform Xbp1s. ATF4, ATF6, and Xbp1 together initiate transcriptional events to resolve ER stress. However, excessive reactive oxygen species (ROS) and ER stress due to excessive lipid accumulation in the liver can lead to inflammation and hepatocyte death [139]. For instance, increased CYP2E1 expression promotes ROS production and the progression of NAFLD. In contrast, stress-induced activation of nuclear erythroid 2-related factor 2 (NRF2) protects against oxidative stress and the pathogenesis of NAFLD [140]. Furthermore, unresolved ER stress results in the activation of apoptotic transcription factor CCAAT/enhancer binding protein (CHOP). The prolonged activation of IRE1α also leads to the activation of the inflammatory transcription factors c-Jun and NF-κB. Thus, the transcription factors involved in stress-induced responses may contribute to the development of NASH (Table 1).

### 5.1. Xbp1

High caloric stress leads to the splicing and nuclear localization of Xbp1, which in turn transcribes factors that improve protein folding as well as lipogenesis [38]. However, obesity-induced chronic stress limits the nuclear localization of Xbp1 and aggravates ER stress and insulin resistance [141]. The rescue of Xbp1 activity in HFD-fed or *ob*/*ob* mice improves glucose homeostasis and reduces hepatic steatosis, which is associated with reductions in the expression of lipogenic genes [142]. Whether the improvement of hepatic steatosis in these mouse models is a direct outcome of transcriptional regulation by Xbp1 or a secondary consequence of resolved metabolic stress and improved insulin sensitivity remains unclear. Nonetheless, the lipogenic role of Xbp1 is demonstrated using a mouse model with a liver-specific ablation of Xbp1 following WD, whereby the loss of Xbp1 is associated with reduced steatosis but enhanced liver injury and fibrosis with the upregulation of type-I collagen α1 (Colα1), TGFβ1, CHOP, and p-JNK [38,143].

### 5.2. ATF4

In NASH patients, the mRNA expression of ATF4 and CHOP and protein expression of CHOP are significantly elevated compared to liver samples from patients with simple steatosis [40]. ATF4 depletion protects mice from high fructose-induced hepatic steatosis by reducing lipogenesis through the reduced hepatic expression of PPARγ, SREBP1c, ACC, and FASN [144].

### 5.3. ATF6

Hepatic ATF6 knockdown or overexpression of its dominant-negative form by adenovirus in WD-fed mice exacerbates insulin resistance and hepatic steatosis with reduced transcriptional activity of the PPARα/RXR complex. Conversely, overexpression of the cleaved active from of ATF6 protects mice from hepatic steatosis and promotes hepatic FA oxidation. Experiments in hepatocytes have shown that ATF6 promotes hepatic FA oxidation by enhancing PPARα transcriptional activity through direct interaction and activates its downstream targets such as carnitine palmitoyltransferase 1 alpha (CPT1α) and medium-chain acyl-CoA dehydrogenase (MCAD) [145]. Activated ATF6 also interacts with SREBP2 and inhibits SREBP2 target genes in hepatocytes [146].

### 5.4. NRF2

NRF2 is the primary driver of gene expression via the antioxidative response elements (ARE). In response to oxidative damage such as lipid peroxidation and DNA damage, NRF2 increases the transcription of antioxidative factors, including [140,147] NRF2, which suppresses inflammation by preventing the increased transcription of pro-inflammatory cytokines [140]. Specifically, NRF2 interferes with the lipopolysaccharide-induced transcriptional upregulation of IL-6 and IL-1β. Accumulating evidence supports a protective role of NRF2 in NASH [148]. In rats and mice with diet-induced NASH, NRF2 activation improves glucose homeostasis and inhibits hepatic steatosis, inflammation, and fibrosis by decreasing lipid synthesis and upregulating β-oxidation and lipoprotein assembly [35,149]. In contrast, the loss of NRF2 exacerbates hepatic steatosis and accelerates the development of NASH in mice fed an HFD or MCD [150,151]. Mechanistically, the oxidative stress due to the deletion of NRF2 in these mice activates NF-κB and leads to the upregulation of the inflammatory cytokines IL-6 and TNFα.

### 5.5. CYP2E1

Although it is not a transcription factor, it is important to include CYP2E1, which becomes activated following insulin resistance and lipotoxicity [152] and promotes ROS production in the setting of NAFLD [153]. CYP2E1 plays key metabolic roles in gluconeogenesis and fatty acid metabolism. It controls the formation of lactate or glucose from the ketone body acetone [154]. Furthermore, CYP2E1 carries out the omega hydroxylation of fatty acids, increasing lipotoxicity and inflammation [155], which represent major pathophysiological mechanisms in NAFLD progression [156]. The role of CYP2E1 in liver injury was first identified following the alcohol-induced induction of CYP2E1 protein. However, clear differences exist between alcoholic liver disease (ALD)- and NAFLD-induced activation of CYP2E1: while alcohol consumption only stabilizes the CYP2E1 protein without changes in mRNA expression, excessive nutrition increases both protein stability and mRNA abundance [157]. Although the transcriptional regulation of CYP2E1 has been linked to the activities of HNF1α [158], HNF4α [108], SP1 [159], and C/EBP [154], the mechanisms by which obesity and NAFLD exacerbate CYP2E1 activity requires additional studies [153].

## 6. Fibrosis

Fibrosis is the strongest predictor of adverse clinical outcomes for NASH. Fibrogenesis during liver injury is initiated by the activation of HSCs in the liver [160,161]. Established inducers of fibrogenesis and HSC activation include adipocyte enhancer binding protein 1 (AEBP1), AATF, yes-associated protein (YAP), and transforming growth factor beta-(TGFβ)-mediated activation of transcription factors against decapentaplegic homolog (SMAD). In addition to these fibrosis-specific regulators, the main transcriptional regulators of lipid homeostasis (including PPARα and PPARγ) and inflammation (RUNX2 and c-Jun) have also been reported to dictate the fibrotic stage in NASH (Table 1).

### 6.1. TGFβ/SMAD axis

TGFβ is secreted from activated HSC and is a potent inducer of fibrogenesis. Its pro-fibrogenic effect is mainly mediated by the TGFβ receptor (TGFβR)-dependent activation of the SMAD family in HSC: the phosphorylated SMAD2/3 complex binds to SMAD4 and translocates to the nucleus to promote the transcription of fibrogenic genes including Co1α1, Co3α1, smooth muscle alpha 2 actin (αSMA), and TGFβ as well as the production of tissue inhibitor of metalloproteinases (TIMPs) [162], which promote fibrosis by inhibiting matrix degradation [163]. In contrast, Smad7 inhibits the regulation of the TGFβ signaling by recruiting ubiquitin E3 ligases that promote the degradation of TGFβR1 and by recruiting the protein phosphatase PP1C, which inactivates TGFβR1 [164]. The livers of NASH patients as well as a mouse model of NASH exhibit increased nuclear localization of the SMAD2/3/4 complex and the reduced expression of SMAD7, which all together contribute to increased TGFβ, Co1α1, and αSMA [42]. The regulation of SMAD2/3 has also highlighted the role of additional transcription factors in mediating TGFβ-mediated fibrogenesis: the interactions of the transcriptional coactivators CREB binding protein (CBP) and p300 with SMAD2/3 promotes histone acetylation and increased transcriptional activity [165]. Supporting the pathophysiological relevance of this axis, the AMPK-mediated degradation of p300 results in the inhibition of TGFβ/SMAD3-mediated fibrogenesis in HSC [166]. Finally, the transcription factor v-ets avian erythroblastosis virus E26 oncogene homolog 1 (ETS1), which is elevated in a NASH mouse model, enhances TGFβ/SMAD signaling by directly binding to SMAD3 and preventing its ubiquitination and degradation [167].

### 6.2. AEBP1

AEBP1 plays a role in adipogenesis [168,169], myofibroblast differentiation [170], and macrophage cholesterol homeostasis [171]. AEBP1 was identified as a key transcription factor during the transition from simple steatosis to NASH using a co-regulatory network approach, which assessed AEBP1 expression in NASH fibrosis versus other NAFLD histological classes using pairwise comparisons [172]. In support of this database analysis, AEBP1 expression increases in the setting of NASH compared to simple steatosis in the livers of *ApoE^+^*^/*−*^ mice. A recent clinical study demonstrated that AEBP1 is specifically expressed in HSC and at a greater extent in the livers of patients with NASH [43]. The ablation of AEBP1 only in the HSC of mice protects against high fat and high cholesterol diet-induced fibrosis. Mechanistically, AEBP1 activates Wnt signaling by specifically binding frizzled-8 and low-density lipoprotein-related receptor 6, which blocks the PPARγ-dependent inhibition of activated HSC. Another study confirmed that hepatic AEBP1 is directly associated with the degree of steatosis, lobular inflammation, and fibrosis in NASH patients [168]. This study also found that AEBP1 upregulates the expression of genes identified as part of an algorithm-predicted AEBP1-associated NASH co-regulatory network [168]. These target genes include the regulators of fibrosis (*AKR1B10*, *CCDC80*, *DPT*, *EFEMP1*, *ITGBL1*, *LAMC3*, *MOXD1*, *SPP1*, and *STMN2*), ECM production and maintenance (*COL4A2* and *MARCO*), and myofibroblast transition (*ACTA2, COL1A1, COL1A2, SERPINE1* and *PLAU*). Taken together, these findings strongly implicate AEBP1 in the diagnosis and treatment of NASH.

### 6.3. YAP

The Hippo pathway and its effector YAP are particularly important for controlling liver size by regulating proliferation and growth [173]. The expression of YAP is barely detectable in healthy livers of humans and mice but becomes activated in the setting of NASH [45]. YAP is expressed in hepatocytes and activates the expression of proteins that promote fibrosis (ColL1α1, TIMP1, TGFβ2) and inflammation (TNFα, IL-1β), which stimulate the expansion of myofibroblasts and the recruitment of immune cells, exacerbating liver fibrosis [174]. YAP is also activated in Kupffer cells by the lipopolysaccharides (LPS)/TLR4 signaling pathway, where it promotes the development of NASH by enhancing the production of pro-inflammatory cytokines [175]. Further gain and loss of function experiments have shown that the activation of the YAP/transcriptional co-activator with PDZ-binding motif (TAZ) axis leads to the expression of a key matricellular chemokine (CYR61), which stimulates and recruits extrahepatic macrophages to promote liver fibrosis.

### 6.4. PPARα

In addition to beneficial effects on steatosis and inflammation, PPARα agonist treatment also reverses fibrosis by targeting PPARα in HSC, which decreases the expression of fibrogenic factors including Col1α1 and TIMPs and reduces the number of activated HSC. The protective effect of PPARα was further demonstrated by treating fibrotic APOE_2_KI811A mice with the PPARα agonist fenofibrate, which protected mice from NASH by reducing both steatosis and hepatic macrophage accumulation [10]. By contrast, mice with a genetic ablation of PPARα display increased susceptibility to NASH [130,176].

### 6.5. PPARγ

In humans, growth factors activate HSC that display decreased PPARγ expression during the progression of NAFLD to NASH [160]. On the other hand, livers with simple steatosis exhibit increased PPARγ expression. The treatment of rats with NASH with the PPARγ agonist pioglitazone prevents hepatic fibrosis and reduces the expression of TIMPs [177]. Indicating that the inhibition of PPARγ in HSC is responsible for the increased transcription of TIMPs, the overexpression of PPARγ reduces the expression of TIMP1, TIMP2, and alpha smooth muscle actin (αSMA) and reverses hepatic fibrosis. By contrast, the HSC-specific ablation of PPARγ aggravates CCl_4_-induced liver fibrosis and increases αSMA expression [132]. Collectively, these findings clearly link decreased PPARγ activity in HSC to hepatic fibrosis. Accordingly, pioglitazone ameliorates only moderate pericentrilobular fibrosis in rats with no effect on severe bridging fibrosis, which is most likely due to the reduced PPARγ availability for pioglitazone to target under the advanced stages of the disease [178]. On the other hand, the effect of TZDs on fibrosis in humans have been less clear. Unlike in rats, a meta-analysis of TZD effects from eight randomized trials (*n* = 516) on NASH-associated liver fibrosis found pioglitazone to significantly improve fibrosis, particularly in the advanced fibrosis stage with bridging fibrosis and cirrhosis compared to NASH with mild perisinusoidal/periportal fibrosis [179]. This effect could have been independent of PPARγ, as TZDs can bind alternative targets such as the mitochondrial pyruvate carrier [179]. In fact, the inhibition of the mitochondrial pyruvate carrier by a next-generation TZD (MSDC0602) was found to reverse hepatic fibrosis in mice, supporting the mitochondria pyruvate carrier as a relevant treatment target [180,181]. Nonetheless, the relevance of targeting PPARγ for the treatment of advanced fibrosis in humans remains unclear.

### 6.6. RUNX2

Studies have shown that Runx2 acts as a fibrogenic or tumorigenic transcription factor in hepatic fibrosis or hepatocellular carcinoma [182,183]. Runx2 is expressed in the non-parenchymal cells of the liver but not in the hepatocytes. In a mouse model of NAFLD/NASH, Runx2 becomes upregulated in the HSCs during the development of NAFLD [163].

### 6.7. c-Jun

The impact of c-Jun on fibrogenesis depends on the liver cell type. The deletion of c-Jun only in hepatocytes reduces steatosis but increases fibrosis, whereas its deletion in both hepatocytes and non-parenchymal cells protects against MCD-induced fibrosis in mice [142]. This was linked to reductions in the pro-inflammatory cytokine osteopontin (Opn, also known as SPP1), which is an established marker of a regenerative response called the ductular reaction (DR), which is an essential driver of fibrogenesis. Additional investigations using *Opn^−^*^/−^ mice established that c-Jun expression in NPLC promotes NASH-related DR and subsequent fibrosis by upregulating Opn expression [39,184].

## 7. Microbiome Dysbiosis

The contribution of obesity-induced changes in the gut microbiome to the pathogenesis and progression of NAFLD [185] was initially established using germ-free mice and fecal transplant from lean [186] and diet-induced obese mice [187]. Furthermore, the inoculation of germ-free mice with the gut microbiota of obese humans [188] and NASH patients [189] leads to the onset of hepatic steatosis and NASH, respectively. These findings formed the base or microbiota-based therapies for NAFLD such as pre- and probiotics and fecal microbiota transplantation [190,191,192].

The gut microbiota can influence the progression of NAFLD through several pathways, which has been reviewed extensively elsewhere [193]. Briefly, these pathways include changes in gut permeability, low-grade inflammation and immune balance, the modulation of dietary choline and bile acid metabolism, and the production of endogenous substrates [186]. In this review, we highlight that the microbiota, through the production of endogenous substrates, may alter the transcriptional profile of the liver. Major metabolites that are linked to alterations in the gut microbiota include bile acids [194,195,196], short-chain fatty acids (SCFA) [197] and lipopolysaccharides (LPS) [189]. These metabolites can play an important role in NAFLD progression by mediating the gut–liver axis [198]. Products derived from bile acid metabolism act on FXR to decrease hepatic triglyceride levels and improve glucose metabolism [90]. Specifically, the HFD-induced remodeling of the gut microbiota increases the production of bile salt hydrolase (BSH), which is a bacterial enzyme that hydrolyzes and inactivates tauro-β-muricholic acid (T-β-MCA) [199]. T-β-MCA inhibits intestinal FXR signaling, which suppresses ceramide synthesis [200]. Therefore, microbiome dysbiosis results in increased FXR signaling and ceramide production, which in turn promotes SREBP1c activity and steatosis in the liver [201].

SCFAs have been shown to increase the AMPK activity in liver and muscle tissue [202]. The activation of AMPK triggers PGC-1α expression, which controls the transcriptional activity of PPARα, PPARγ, PPARδ, LXR, and FXR, which are important transcriptional regulators of cholesterol, lipid, and glucose metabolism [203]. LPS has been shown to activate NF-κB in cultured hepatocytes [204], which plays a major role in the development of inflammation during NAFLD progression [91] and is highly activated in both mice and patients with NASH. Furthermore, LPS can induce MAP kinase kinase-3 (MKK3) activation, which in turn stimulates C/EBPβ and C/EBPδ binding elements to promote the transcription of CYP2E1 and induce oxidative stress [154].

## 8. Prediction of Transcriptional Regulators by Database Analyses

### 8.1. Prognostic Biomarkers for Human NAFLD and NASH

Many transcriptomic studies have been conducted to elucidate novel biomarkers for the different stages of NAFLD, including steatosis, ballooning, and fibrosis. To elucidate the transcriptional changes that are associated with human NAFLD, we procured publicly available human NAFLD/NASH transcriptome data from the Gene Expression Omnibus (GEO) and subjected them to Ingenuity Pathway Analysis for the prediction of changes in upstream factors (Table 2). Predictions were based on two GEO datasets with strong power analysis (Table 2) as well as a previously published Ingenuity Pathway Analysis (IPA)-based prediction analysis [7,205,206]. The activation of PPARγ was the only consistent prediction for simple steatosis, whereas the onset of fibrosis was associated with changes in a larger number of transcription factors, which were consistent in at least half of the datasets. These included the activation of inflammation (NF-κB, RELA, JUN, IRF1, IRF3, STAT1, SP1), glucose production (FOXO1), and lipogenesis (SREBP1), as well as the inhibition of PPARα, PPARγ, and RXRα. The activation of C/EBPβ, CTNNB1, and SMAD3 and the inhibition of HNF4α and SMAD7 were also associated with NASH and NASH-induced HCC, suggesting that these factors might contribute to the pathogenesis of advanced stage fibrosis.

### 8.2. Altered Transcription Factors in Mouse Models of NAFLD and NASH

The same strategy was applied to predict the changes in the activity of transcription factors in mouse models of NAFLD/NASH. We performed IPA upstream activity prediction analysis for publicly available liver transcriptome data from mouse models of steatosis that were established by feeding high caloric diets [18,207] as well as mouse models of NASH that were established by feeding an MCD diet [207], NASH diet, or a combination of high caloric diets coupled with CCl_4_ treatment [18,208]. Additionally, we included the observations of a previously published STZ-induced NASH and HCC model (STAM) [7]. Transgenic mouse models that involve genetic manipulations were excluded, since our comparative analysis was not aimed at delineating the transcriptional consequences of rare gene variants in humans and mice. As anticipated, the activation of most consistent pathways in humans (Table 2) were also confirmed in most mouse models. However, in contrast to the downregulation of PPARα, PPARγ, and RXRα in human livers with NASH, these pathways were upregulated in most mouse models of NASH (Table 2). The activity regulation of PPARα, PPARγ, and RXRα in mouse models of NASH was instead more representative of their activity in human livers with simple steatosis (Table 2).

IPA analysis of the mouse models also identified pathways that were not predicted to be affected among human NASH datasets. Among these, the activation of SMAD2, SMAD4, YAP1, NOTCH1, EP300, p63, and the inhibition of nuclear receptor corepressor (NCOR) has been previously linked to human NASH by independent studies (Table 3) [42,166,173,209,210,211,212]. Therefore, most mouse models mimic the transcriptional signature of human NASH for these transcription factors. However, the consistent inhibition of SREBP2 and the activation of fos proto-oncogene (FOS) and PGC1α in mouse NASH models mimicked human steatosis but were absent in the setting of human fibrosis (Table 3) [102,213,214]. Furthermore, increased FOS mRNA in fibrotic versus non-fibrotic NAFLD patients was in line with increased EGR1 activity, which promotes FOS expression, as previously published (Table 2). However, IPA did not predict FOS activation, as this would be anticipated to inhibit NF-κB and SP1 and activate CEBBP, which was in contrast with the regulation in fibrotic livers (Table 2). Other frequently altered transcriptional mechanisms in mouse NASH models, which were not previously associated with human NASH, included PPARδ, HIF1α, MED1, NCOA1, NCOA2, SMARCA4, FOXO3, HDAC2, STAT5b, and STAT6 (Table 4). Individual transcriptomic datasets from mouse livers also predicted the regulation of unique pathways for each dataset, which were not predicted to be regulated in other mouse models (Table 4). Since IPA prediction of upstream factors in human NASH also failed to identify changes in the activity of these sets of transcription factors in Table 3 and Table 4, studying their relevance in the pathogenesis of NASH in humans would be beneficial prior to investigating their roles in pre-clinical rodent models of NASH. It is worth mentioning that the activation of ChREBP and C/EBPα were only confirmed in the mouse models using MCD and WD coupled with CCl_4_, respectively, but they were not detected in human fibrosis datasets. Another category of altered transcription factors belonged to those that were altered in a single human dataset but not in any of the mouse models (Table 5). Although the activity regulation of these factors could be relevant in the pathogenesis of NASH, corroborating evidence is lacking.

To determine how the transcriptional activity of popular NASH mouse models faired against human NASH, we implemented a scoring strategy ranging from +2 to −2 for each transcription factor: +2 for the confirmation of a transcriptional activity in a mouse model, which was observed in more than one human NASH dataset (i.e., NF-κB); −2 for the reversal of a transcriptional activity, which was observed in more than one human NASH dataset (i.e., PPARγ); +1 for the confirmation of a transcriptional activity, which was observed only in one human NASH dataset (i.e., SMAD7); −1 for the reversal of a transcriptional activity, which was observed only in one human NASH dataset (i.e., THRβ for CCl_4_ model); −1 when multiple confirmations for a transcriptional activity among human datasets remained unchanged in a mouse model (i.e., NF-κB for NASH diet + CCl_4_ model); 0 for a lack of transcriptional activity in a mouse model, which was also observed in some of the human NASH datasets (i.e., THRb for all models except CCl_4_); and 0 when a transcriptional activity was predicted to be inconsistently regulated among different human NASH datasets regardless of the state of the activity of that transcription factor within the mouse models (i.e., STAT3). From a possible maximum score of +47 for all the common transcription factors (Table 2), the NASH diet, MCD diet, NASH diet + CCl_4_, CCl_4_ alone, and WD + CCl_4_ netted total scores of 23, 27, −10, 12 and 7, respectively, suggesting that the NASH diet and MCD diet exhibit transcriptional activity profiles that are more representative of human NASH, whereas the models that involved CCl_4_ treatment did not. We excluded the STAM mouse model due to the low number of predicted matches.

## 9. Conclusions

Maladaptive responses to obesity results in the activation of inflammatory and fibrogenic pathways in the liver. Here, we reviewed the transcription factors, the activity of which have been commonly associated with obesity-induced NAFLD and NASH. The development of NAFLD and NASH strongly correlates with the dysregulation of transcriptional regulators that play a role in lipid metabolism, inflammation, metabolic stress, and fibrosis. Interestingly, the review of gluconeogenic transcription factors indicated a protective function against steatosis and NASH, since their loss often resulted in disease. The field of main regulators will continue to increase with heightened focus on delineating new pathways in the pathogenesis of NAFLD, as each of the areas discussed in this review are still being actively researched and adding to our understanding of the transcriptional regulation of NAFLD.

Our review also indicates that none of the diet-based rodent models replicate all the features of the human pathophysiology. Our observations suggested that the FCP diet and MCD diet exhibit transcriptional activity profiles that are more representative of human NASH, whereas the models that involved chemical induction, such as CCl_4_ treatment, did not. The generation of novel experimental models that more accurately reproduce human pathophysiology, including mice with humanized livers [215], will be central to the discovery of tractable targets for the management of NAFLD.

## Figures and Tables

**Table 1:** Changes in the activity of transcription factors that regulate glucose and lipid metabolism, inflammation and fibrosis in the setting of NAFLD in humans and mice.

Factor	Model	Pathway	Regulation	Reference
PPARα	Humans, mice	Lipid metabolism, inflammation, fibrosis	Upregulation	[20]
PPARγ	Humans, mice	Lipid metabolism, inflammation, fibrosis	Upregulation	[20]
SREBP Family	Humans, mice	Lipid metabolism	Genetic variations increase risk of NAFLD	[21]
ChREBP	Humans, mice	Lipid metabolism	Upregulation	[22]
CAR	Humans, mice	Lipid metabolism, inflammation	Upregulation	[23]
LXR	Humans	Lipid metabolism, inflammation	Upregulation	[24]
FXR	Humans	Lipid metabolism	Downregulation	[25]
STAT5	Humans	Lipid metabolism	Upregulated	[26]
C/EBPα	Mice	Lipid metabolism	Upregulation	[27]
PGC1α	Mice	Glucose homeostasis	Downregulation	[28]
FoxO	Humans	Glucose homeostasis	Upregulation	[29]
HNF4α	Humans	Central regulator, Glucose homeostasis	Downregulation	[25]
NF-κB	Humans, mice	Inflammation	Upregulation	[30]
IRFs	Mice	Inflammation	Upregulation	[31]
STAT1/3	Mice	Inflammation	Upregulation	[32]
AP-1 and c-Jun	Humans, mice	Inflammation, fibrosis	Upregulation	[30,33]
SHP	Humans, mice	Inflammation	Downregulation	[34]
Nrf2	Mice	Inflammation	Upregulation	[35]
Runx2	Mice	Inflammation	Upregulation	[36]
C/EBPβ		Inflammation		
IRE1α	Human	Metabolic stress	Upregulation	[37]
Xbp1	Mice	Metabolic stress	Upregulation	[38]
eIF2α	Mice	Metabolic stress	Upregulation	[39]
ATF4	Humans	Metabolic stress	Upregulation	[40]
ATF6	Humans	Metabolic stress	Upregulation	[41]
Smad	Humans, mice	Fibrosis	Upregulation	[42]
TGFβ	Humans, mice	Fibrosis	Upregulation	[42]
AEBP1	Humans, mice	Fibrosis	Upregulation	[43]
AATF/che-1	Humans, mice	Fibrosis	Upregulation	[44]
YAP	Humans, mice	Fibrosis	Upregulation	[45]

Abbreviations: peroxisome proliferator-activated receptor (PPAR), sterol regulatory element binding protein (SREBP), carbohydrate-responsive element-binding protein (ChREBP), constitutive androstane receptor (CAR), liver X receptor (LXR), farnesoid X receptor (FXR), signal transducer and activator of transcription (STAT), CCAAT/enhancer binding protein (C/EBP), PPARγ coactivator 1 alpha (PGC1α), forkhead protein O (FoxO), hepatocyte nuclear factor (HNF), nuclear factor of the κ light chain enhancer of B cells (NF-κB), interferon regulatory factors (IRFs), activator protein 1 (AP-1), small heterodimer partner (SHP), nuclear factor erythroid 2-related factor 2 (Nrf2), runt-related transcription factor 2 (Runx2), inositol-requiring enzyme 1α (IRE1α), X box-binding protein 1 (Xbp1), eukaryotic translation initiation factor 2α (eIF2α), activating transcription factor (ATF), transcription factors against decapentaplegic homolog (Smad), transforming growth factor β (TGFβ), adipocyte enhancer binding protein 1 (AEBP1), apoptosis antagonizing transcription factor (AATF/che-1), yes-associated protein (YAP).

**Table 2:** Ingenuity Pathway Analysis (IPA) prediction of upstream mechanistic networks that are commonly regulated in the livers of humans and mice with NAFLD.

		Human							Mouse Model							
		Steatosis		Fibrosis				HCC	Steatosis		NASH					
Regulation in Human Fibrosis	Transcription Factor/Regulator	^c^ Non-fibrotic NAFLD vs. Healthy	^e^ Steatotic vs. Healthy	^a^ Fibrotic vs. Healthy	^b^ Fibrotic vs. Non-fibrotic NAFLD	^d^ NASH vs. Healthy	^f^ External IPA NASH	^g^ External IPA HCC	^h^ HFD	^i^ WD	^j^ MCD	^k^ NASH Diet	^l^ NASH Diet + CCl_4_	^n^ CCl_4_	^m^ WD + CCl_4_	^o^ STAM
Consistent Activation (2 ≥ datasets)	FOXO1 *															
	IRF1 *															
	IRF3 *															
	JUN *															
	NFκB *															
	RELA *															
	SP1 *															
	SREBP1 *															
	STAT1 *															
	C/EBPβ ^†^															
	CTNNB1 ^†^															
	SMAD3 ^†^															
Activation (1 dataset)	CREB															
	EGR1															
	ESR2															
	IRF7															
	LXR															
	NFAT															
	NRF2															
	RARα															
	RUNX2															
	SPI1															
	STAT2															
Consistent Inhibition (2 ≥ datasets)	PPARα *															
	PPARγ *															
	RXRα *															
	HNF4α ^†^															
	SMAD7 ^†^															
Inhibition (1 dataset)	AHR															
	HDAC1															
	HNF1α															
	NR1h															
	NR3C1															
	NR5A2															
	RBL1															
	STAT5a															
	THRβ															
Inconsistent Regulation	STAT3															
	MYC															
	p53															
	IRF8															
	ESR1															

Human GEO Accession GSE130970 was divided into three independent IPA comparative analyses: ^a^ Advanced fibrotic (fibrosis score > 3, *n* = 16) versus healthy (*n* = 8), ^b^ Fibrotic versus non-fibrotic NAFLD (NAS > 3, *n* = 11), and ^c^ non-fibrotic NAFLD versus healthy. Human GEO Accession GSE89632 was similarly analyzed in three independent analyses comparing transcriptomic changes among human livers with ^d^ NASH (*n* = 19) vs. healthy (*n* = 24), NASH vs. simple steatosis (*n* = 20) and ^e^ simple steatosis vs. healthy. The conclusions of a previously published upstream regulators analysis for ^f^ NASH and ^g^ NASH-associated HCC are included as well. The mouse models of steatosis using ^h^ HFD (GSE93132) and ^i^ Western diet (GSE99010) as well as mouse models of NASH using ^j^ MCD diet (GSE93132), ^k^ NASH diet (GSE52748), ^l^ NASH diet coupled with CCl_4_ treatment (GSE129525), ^m^ CCl_4_ treatment alone (GSE99010) and ^n^ Western diet coupled with CCl_4_ treatment (GSE99010). The observations from the ^o^ STAM NASH model are included as well. Transcriptomic changes of 2-fold or more were included in the IPA. The table includes the mechanistic network of the transcription factors with a prediction *Z* score of greater than 2 for activation (red) or less than −2 for inhibition (blue). Datasets for mouse hepatocellular carcinoma (HCC) models using extended CCl_4_ treatment for 24 weeks were excluded from the IPA prediction. The analysis of human livers with NASH vs. simple steatosis from GSE89632 dataset did not yield any prediction for significant changes in upstream activity and was excluded from the table. Abbreviations: forkhead protein O (FoxO), interferon regulatory factor (IRF), nuclear factor of the κ light chain enhancer of B cells (NF-κB), v-rel avian reticuloendotheliosis viral oncogene homolog A (RELA), Specific Protein 1 (SP1), sterol regulatory element binding protein (SREBP), signal transducer and activator of transcription (STAT), CCAAT/enhancer binding protein (C/EBP), Catenin Beta 1 (CTNNB1), transcription factors against decapentaplegic homolog (SMAD), cyclic AMP-responsive element-binding protein (CREB), early growth response 1 (EGR1), estrogen receptor (ESR), liver X receptor (LXR), nuclear factor of activated T-cells (NFAT), nuclear factor. * Consistent regulation in 2 or more human NASH datasets. ^†^ Consistent regulation in human NASH and HCC.

**Table 3:** IPA prediction of upstream regulators that were detected in the livers of mouse models of NASH but not in human NASH cohorts.

	^j^ MCD	^k^ NASH Diet	^l^ NASH Diet + CCl_4_	^n^ CCl_4_	^m^ WD + CCl_4_	Association with Human Fibrosis
SMAD4						NASH
SMAD2						NASH
YAP1						NASH
NOTCH1						NASH
EP300						NASH
NCOR						NASH
p63						NASH, steatosis
SREBP2						Steatosis
CAR						Steatosis
FOS						Steatosis, insulin resistance
PGC1α						Steatosis, insulin resistance
PPARδ						N/A
HIF1α						N/A
MED1						N/A
NCOA1						N/A
SMARCA4						N/A
NCOA2						N/A
FOXO3						N/A
HDAC2						N/A
STAT5b						N/A
STAT6						N/A

Mouse models of NASH using ^j^ MCD diet (GSE93132), ^k^ NASH diet (GSE52748), ^l^ NASH diet coupled with CCl_4_ treatment (GSE129525), ^m^ CCl_4_ treatment alone (GSE99010) and ^n^ Western diet coupled with CCl_4_ treatment (GSE99010). Abbreviations: transcription factors against decapentaplegic homolog (SMAD), yes-associated protein (YAP), notch receptor 1 (NOTCH1), Histone Acetyltransferase P300 (EP300), nuclear receptor corepressor (NCOR), sterol regulatory element binding protein (SREBP), constitutive androstane receptor (CAR), fos proto-oncogene (FOS), PPARγ coactivator 1 alpha (PGC1α), peroxisome proliferator-activated receptor (PPAR), hypoxia inducible factor 1α (HIF1α), mediator complex subunit 1 (MED1), nuclear receptor coactivator (NCOA), SWI/SNF related, matrix associated, actin dependent regulator of chromatin subfamily A member 4 (SMARCA4), forkhead protein O (FoxO), histone deacetylase (HDAC), signal transducer and activator of transcription (STAT).

**Table 4:** IPA prediction of upstream mechanistic networks that are unique to each mouse model.

	^j^ MCD	^k^ NASH Diet	^l^ NASH Diet + CCl_4_	^n^ CCl_4_	^m^ WD + CCl_4_
AR					
ARNTL					
CDKN2A					
C/EBPα					
ChREBP					
CIITA					
E2F					
FOXO4					
HNF1α					
HSF1					
IRF9					
KLF4					
MAX					
MYB					
PGR					
RARB					
RB1					
RORA					
SNAI					
SP3					
STAT4					
TCF7L2					
THRα					
VDR					
WT1					
Ybx1					
ZNFn1a1					

Mouse models of NASH using ^j^ MCD diet (GSE93132), ^k^ NASH diet (GSE52748), ^l^ NASH diet coupled with CCl_4_ treatment (GSE129525), ^m^ CCl_4_ treatment alone (GSE99010) and ^n^ Western diet coupled with CCl_4_ treatment (GSE99010). Abbreviations: androgen receptor (AR), aryl hydrocarbon receptor nuclear translocator like (ARNTL), cyclin dependent kinase inhibitor 2A (CDKN2A), CCAAT/enhancer binding protein (C/EBP), class ii major histocompatibility complex transactivator (CIITA), forkhead protein O (FoxO), hepatocyte nuclear factor (HNF), heat shock transcription factor 1 (HSF1), interferon regulatory factor (IRF), kruppel like factor 4 (KLF4), MYC associated factor X (MAX), myb proto-oncogene (MYB), progesterone receptor (PGR), retinoic acid receptor beta (RARB), retinoblastoma transcriptional corepressor 1 (RB1), retinoic acid receptor-related orphan receptor alpha (RORA), snail family transcriptional repressor (SNAI), specificity protein 3 (SP3), signal transducer and activator of transcription (STAT), transcription factor 7 like 2 (TCF7L2), thyroid hormone receptor α (THRα), vitamin D receptor (VDR), Wilms’ tumor protein (WT1), Y box-binding protein 1 (Ybx1), IKAROS family zinc finger 1 (ZNFn1a1).

**Table 5:** IPA prediction of upstream mechanistic networks that are unique to each human NASH cohort.

	^a^ Fibrotic vs. Healthy	^b^ Fibrotic vs. Non-Fibrotic NAFLD	^d^ NASH vs. Healthy	^f^ External IPA NASH
CCND1				
CCNE1				
HMGB1				
IRF2				
IRF5				
KLF2				
NRIP1				
SOX2				

Human GEO Accession GSE130970 was divided into three independent IPA comparative analyses: ^a^ Advanced fibrotic (fibrosis score > 3, n = 16) versus healthy (n = 8), ^b^ Fibrotic versus non-fibrotic NAFLD (NAS > 3, n = 11), and ^d^ non-fibrotic NAFLD versus healthy. ^f^ Previously reported IPA of NASH (Kakehashi et al.). Abbreviations: cyclin D1 (CCND1), cyclin E1 (CCNE1), high mobility group box 1 (HMGB1), interferon regulatory factor (IRF), kruppel like factor 2 (KLF2), nuclear receptor interacting protein 1 (NRIP1), sex determining region Y box transcription factor 2 (SOX2).
